# Supplementary material for: Application of machine learning models for property prediction to targeted protein degraders
Source: Nat Commun. 2024 Jul 9;15:5764. doi: 10.1038/s41467-024-49979-3 (PMC11233499; doi:10.1038/s41467-024-49979-3)
Supplement: Supplementary file 1 — Supplementary Information [file 41467_2024_49979_MOESM1_ESM.pdf]

Supplementary Information

Application of machine learning models for property prediction to targeted protein degraders

Giulia Peteani[1], Minh Tam Davide Huynh[1], Grégori Gerebtzoff[1], and Raquel Rodríguez-Pérez[1]*

[1]Novartis Biomedical Research, Novartis Campus, 4002 Basel, Switzerland

*Corresponding author

R.R.P. Phone: 41-795-42-2309, E-mail: raquel.rodriguez_perez@novartis.com

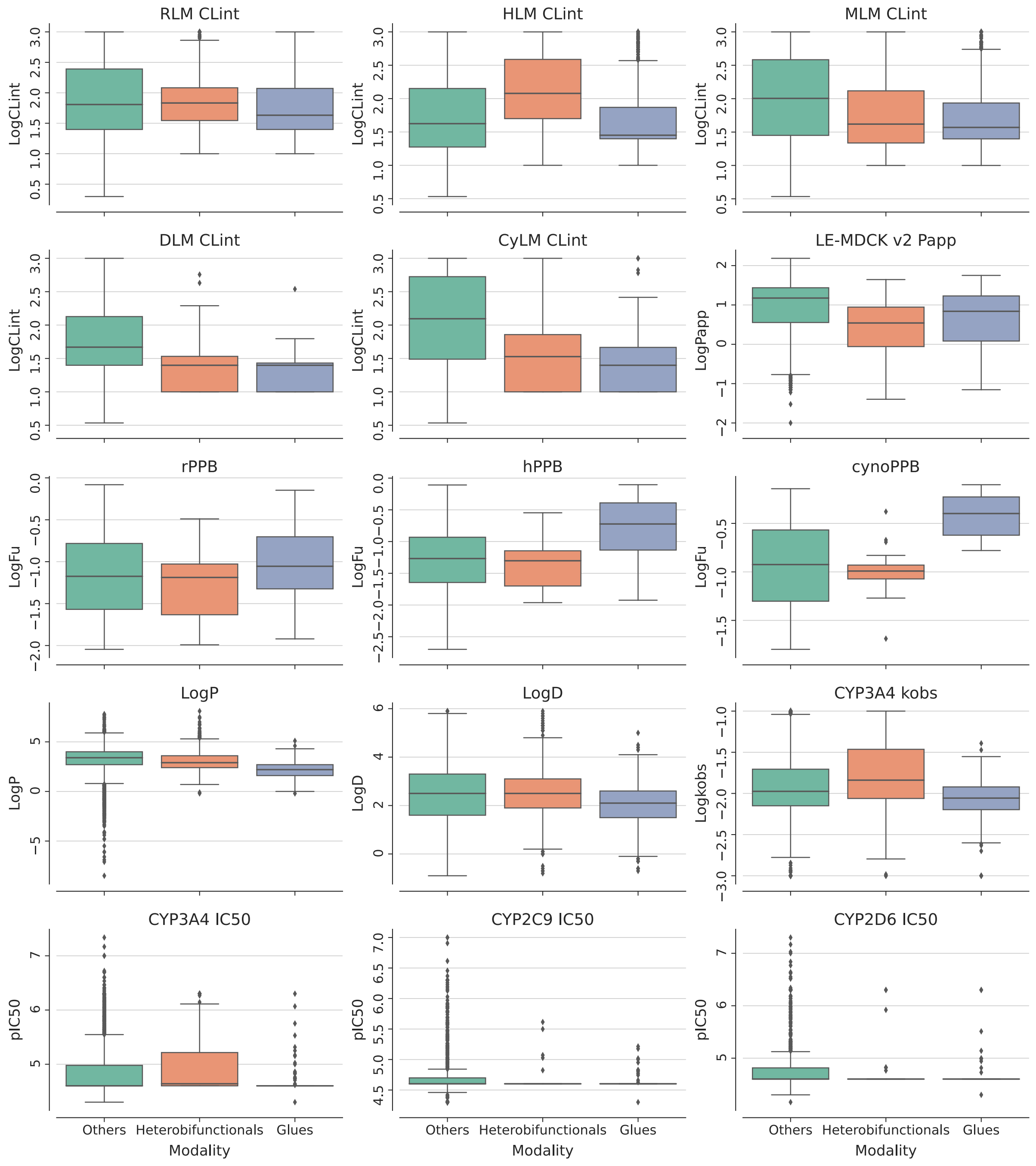

Figure S1. Distribution of experimental assay values. Reported are assay value distributions for the absorption, distribution, metabolism, and excretion (ADME) assays evaluated in this work: permeability (LE-MDCK v2 P_{app}), metabolic clearance (CL_{int}, in rat, human, mouse, dog, and cynomolgus monkey liver microsomes), PPB for rat, human, and monkey, lipophilicity (LogP and LogD), and CYP inhibition (TDI of CYP3A4 and reversible inhibition of CYP3A4, CYP2C9, and CYP2D6). Colors indicate the drug modality: glues (blue) or

heterobifunctionals (orange), or other modalities (green). Boxplots show the median (center line), and 1st and 3rd quartiles (Q_1 and Q_3, respectively) values. Datapoints below Q_1 – (1.5*IQR) or above Q_3 + (1.5*IQR) are considered outliers. Assays are described in Table 1 and Figure 1. Source data are provided as a Source Data file.

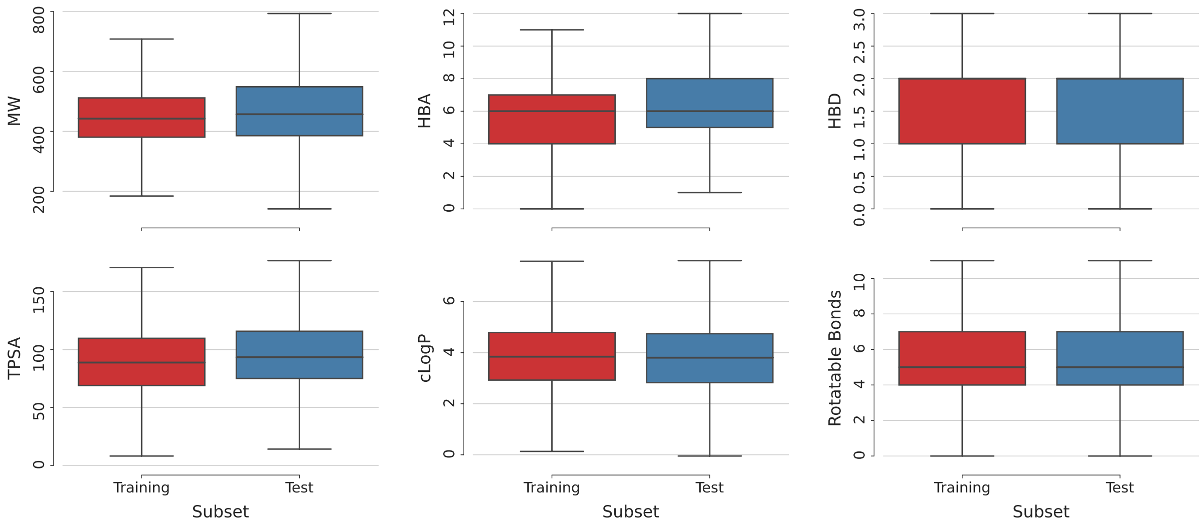

Figure S2. Distribution of calculated properties for training and test sets. The distributions of molecular weight (MW), number of hydrogen bond acceptors (HBA) and donors (HBD), topological polar surface area (TPSA), calculated LogP (cLogP), and number of rotational bonds are reported for the training (red) and test (blue) sets. Boxplots show the median (center line), 1st and 3rd quartile (Q_1 and Q_3, respectively) values. Datapoints below $Q_1 – (1.5*IQR)$ or above $Q_3 + (1.5*IQR)$ are considered outliers and not shown in the boxplots. Training and test set cardinalities are described in Figure 1. Source data are provided as a Source Data file.

(A)

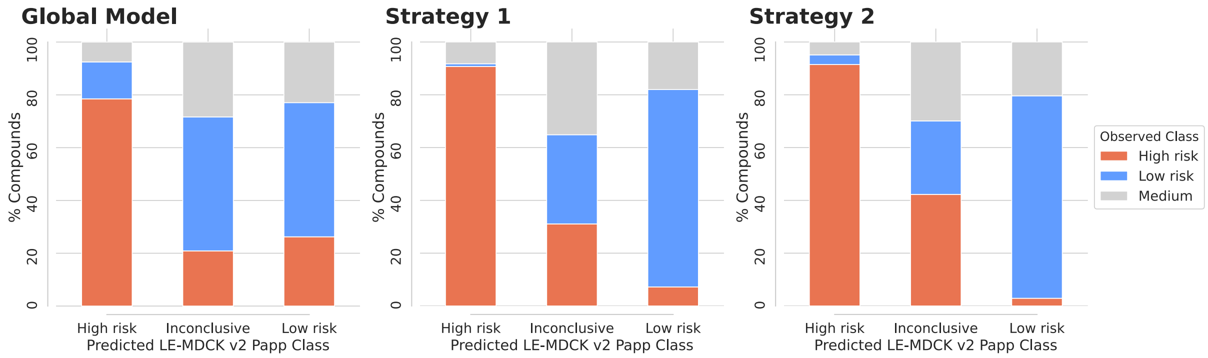
Global Model
Strategy 1
Strategy 2
% Compounds
0
20
40
60
80
100
High risk
Inconclusive
Low risk
Predicted LE-MDCK v2 Papp Class
Observed Class
High risk
Low risk
Medium

(B)

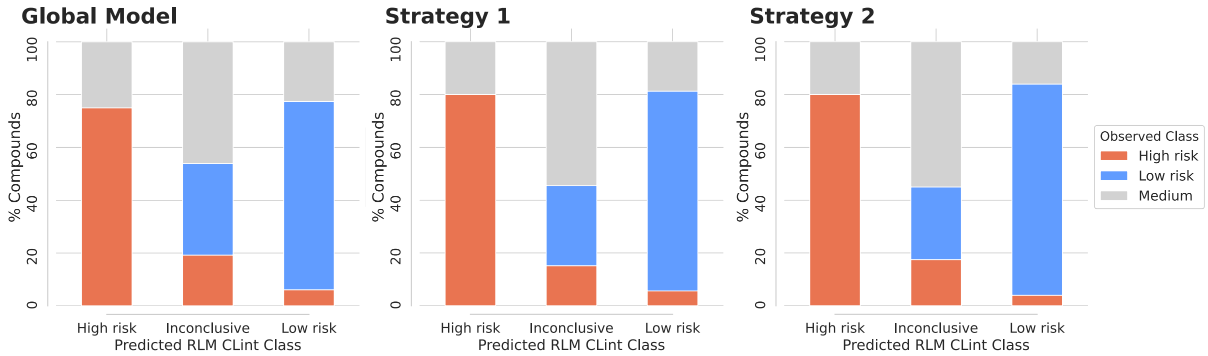
Global Model
Strategy 1
Strategy 2
% Compounds
0
20
40
60
80
100
High risk
Inconclusive
Low risk
Predicted RLM CLint Class
Observed Class
High risk
Low risk
Medium

(C)

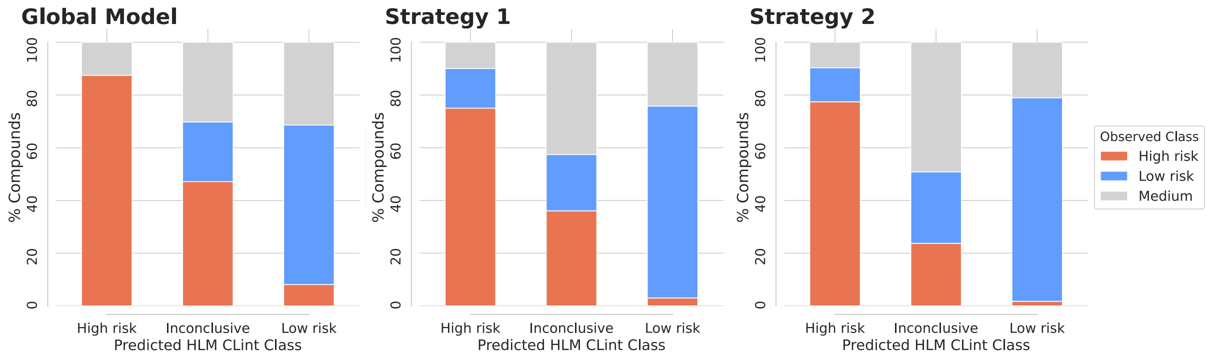
Global Model
Strategy 1
Strategy 2
% Compounds
0
20
40
60
80
100
High risk
Inconclusive
Low risk
Predicted HLM CLint Class
Observed Class
High risk
Low risk
Medium

(D)

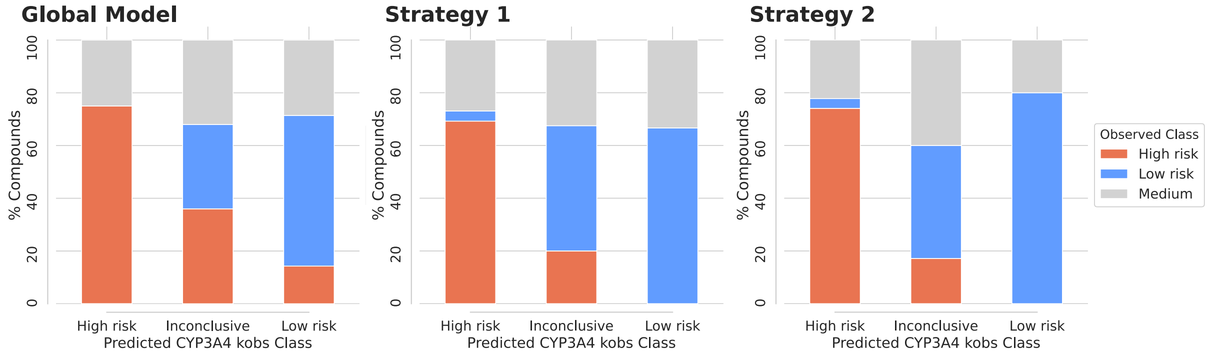
Global Model
Strategy 1
Strategy 2
% Compounds
0
20
40
60
80
100
High risk
Inconclusive
Low risk
Predicted CYP3A4 kobs Class
Observed Class
High risk
Low risk
Medium

(E)

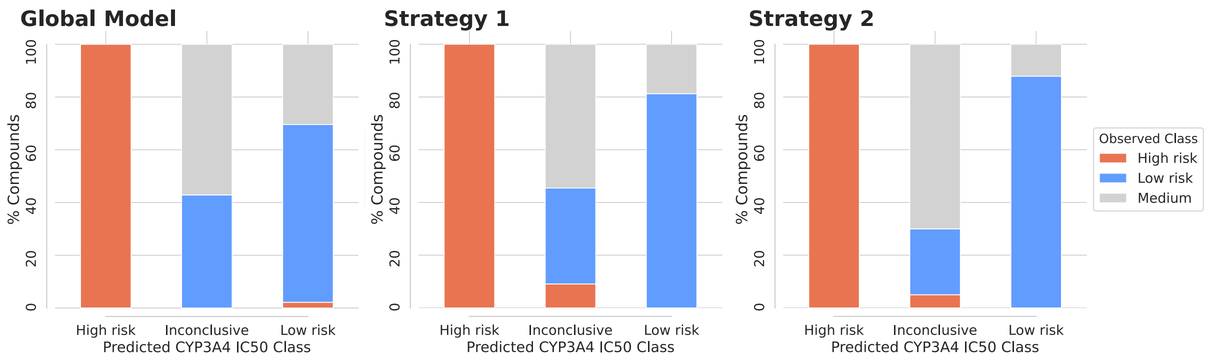

Figure S3. Classification results for heterobifunctional targeted protein degraders (TPDs) with global models and fine-tuning strategies. Reported are the percentage of compounds (y-axes) that have had a given prediction (x-axes) by the original global ML models and the fine-tuned models with two strategies. Strategy I updates model weights with all new data (compounds registered and measured in 2022), whereas strategy II utilizes all heterobifunctional TPDs' assay data for refining the model. Colors indicate the experimental 3-class readout. Classification predictions are shown for passive permeability (A; LE-MDCK P_{app}), metabolic clearance in rat liver microsomes (B; RLM CL_{int}) and human liver microsomes (C; HLM CL_{int}), CYP3A4 TDI (D; CYP3A4 k_{obs}) and reversible inhibition (E; CYP3A4 IC_{50}). Assays are described in Table 1. Source data are provided as a Source Data file.

(A)

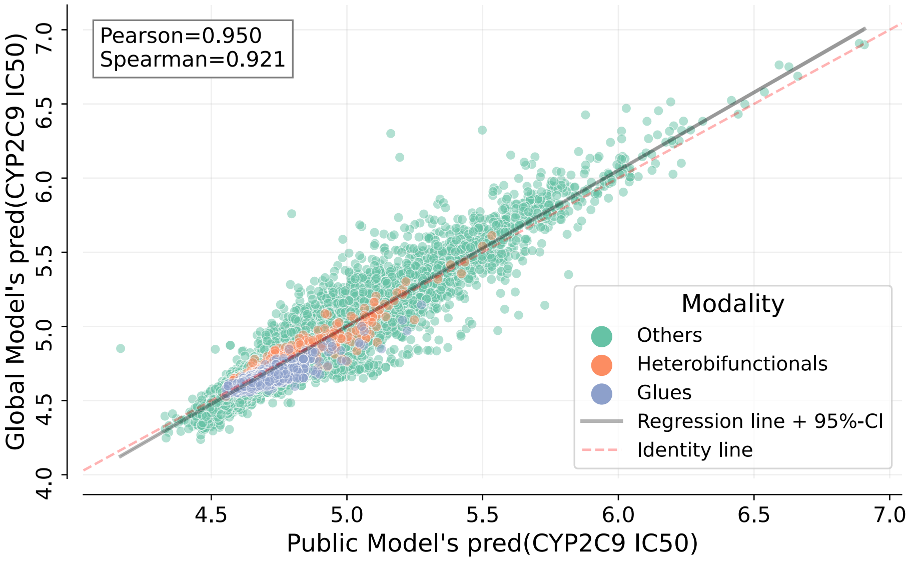

Pearson=0.950
Spearman=0.921
Modality
Others
Heterobifunctionals
Glues
Regression line + 95%-CI
Identity line
Global Model's pred(CYP2C9 IC50)
4.0
4.5
5.0
5.5
6.0
6.5
7.0
4.5
5.0
5.5
6.0
6.5
7.0
Public Model's pred(CYP2C9 IC50)

(B)

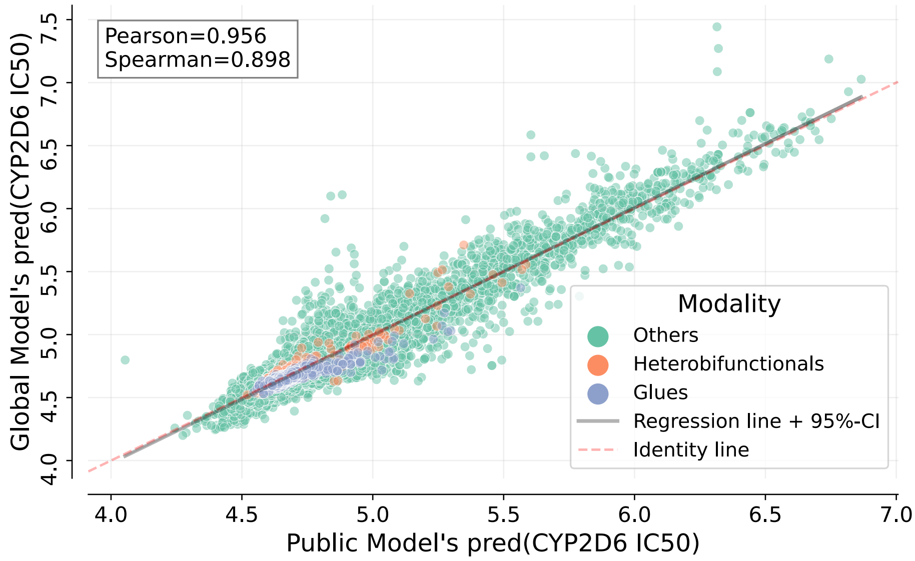

Pearson=0.956
Spearman=0.898
Modality
Others
Heterobifunctionals
Glues
Regression line + 95%-CI
Identity line
Global Model's pred(CYP2D6 IC50)
4.0
4.5
5.0
5.5
6.0
6.5
7.0
7.5
4.0
4.5
5.0
5.5
6.0
6.5
7.0
Public Model's pred(CYP2D6 IC50)

(C)

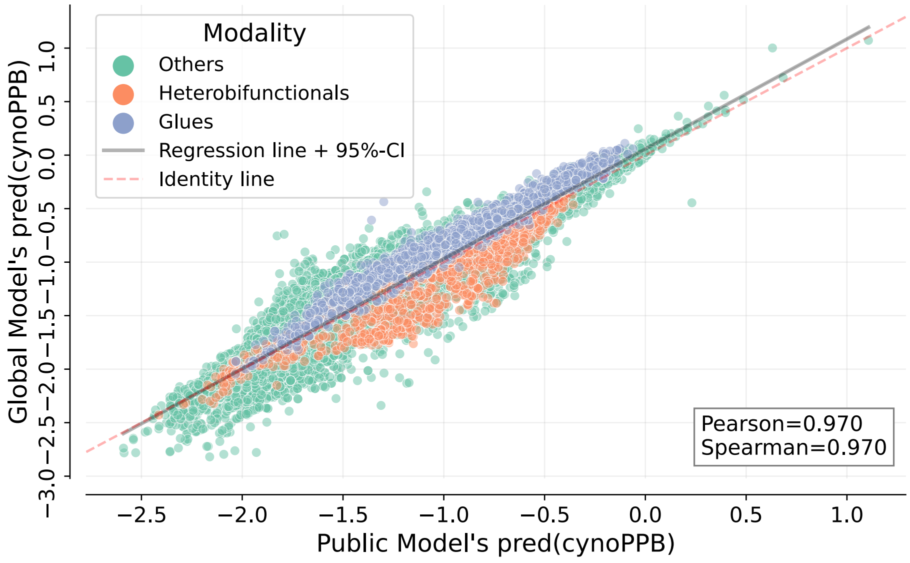

Modality
Others
Heterobifunctionals
Glues
Regression line + 95%-CI
Identity line
Pearson=0.970
Spearman=0.970
Global Model's pred(cynoPPB)
−3.0
−2.5
−2.0
−1.5
−1.0
−0.5
0.0
0.5
1.0
−2.5
−2.0
−1.5
−1.0
−0.5
0.0
0.5
1.0
Public Model's pred(cynoPPB)

(D)

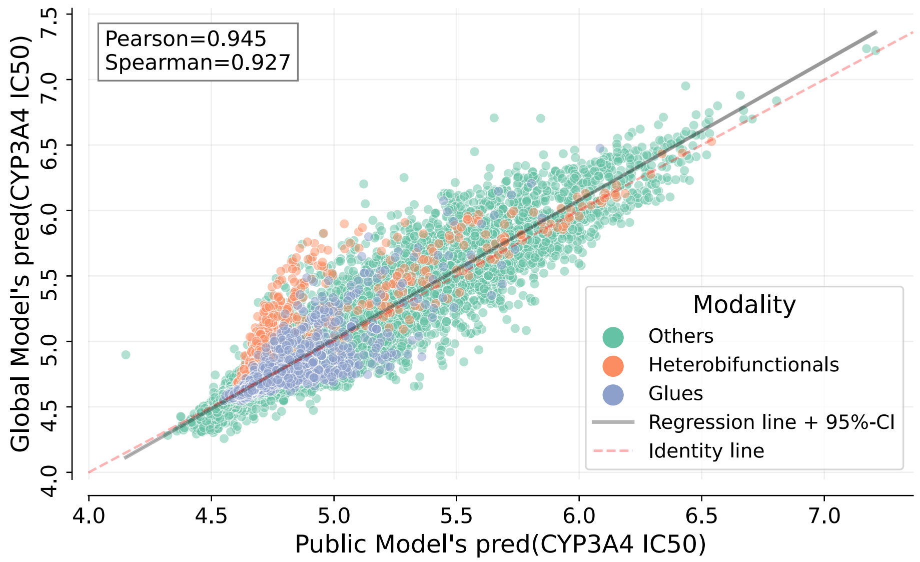
Pearson=0.945
Spearman=0.927
Modality
Others
Heterobifunctionals
Glues
Regression line + 95%-CI
Identity line
Global Model's pred(CYP3A4 IC50)
Public Model's pred(CYP3A4 IC50)
4.0
4.5
5.0
5.5
6.0
6.5
7.0
7.5

(E)

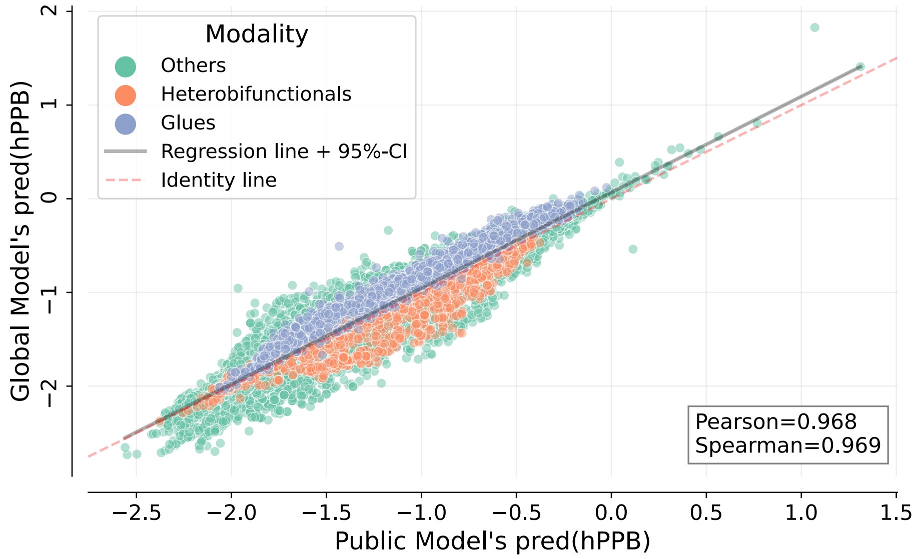
Modality
Others
Heterobifunctionals
Glues
Regression line + 95%-CI
Identity line
Pearson=0.968
Spearman=0.969
Global Model's pred(hPPB)
Public Model's pred(hPPB)
−2.5
−2.0
−1.5
−1.0
−0.5
0.0
0.5
1.0
1.5

(F)

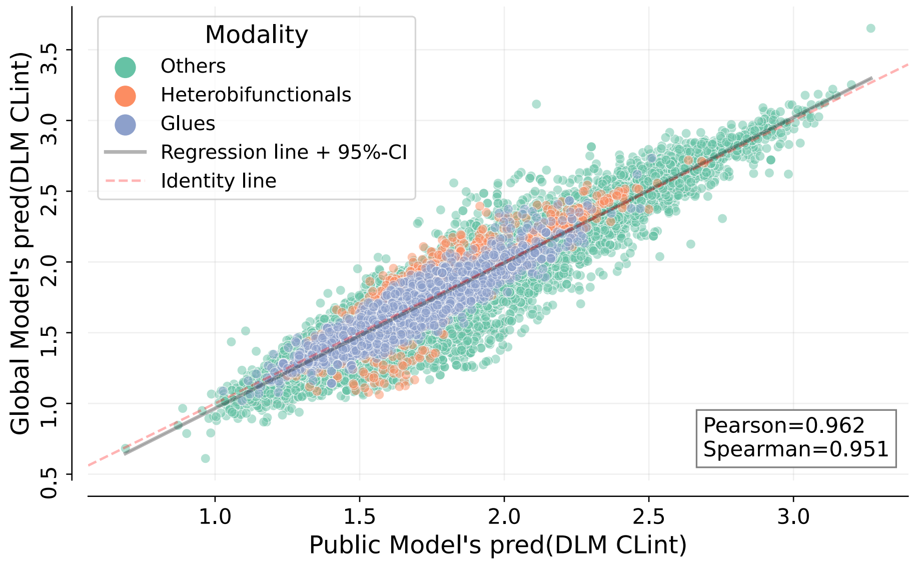
Modality
Others
Heterobifunctionals
Glues
Regression line + 95%-CI
Identity line
Pearson=0.962
Spearman=0.951
Global Model's pred(DLM CLint)
Public Model's pred(DLM CLint)
0.5
1.0
1.5
2.0
2.5
3.0
3.5

(G)

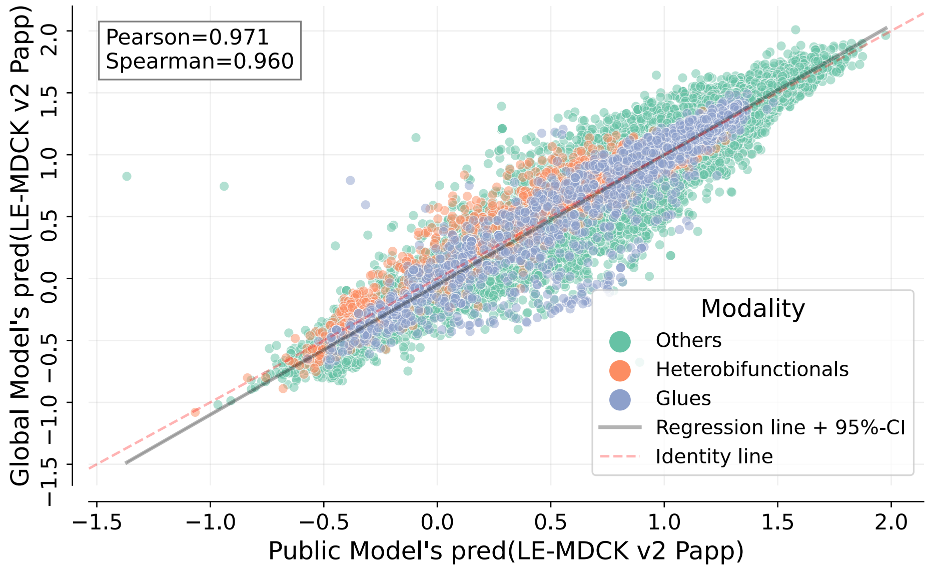

Pearson=0.971
Spearman=0.960
Modality
Others
Heterobifunctionals
Glues
Regression line + 95%-CI
Identity line
Global Model's pred(LE-MDCK v2 Papp)
Public Model's pred(LE-MDCK v2 Papp)

(H)

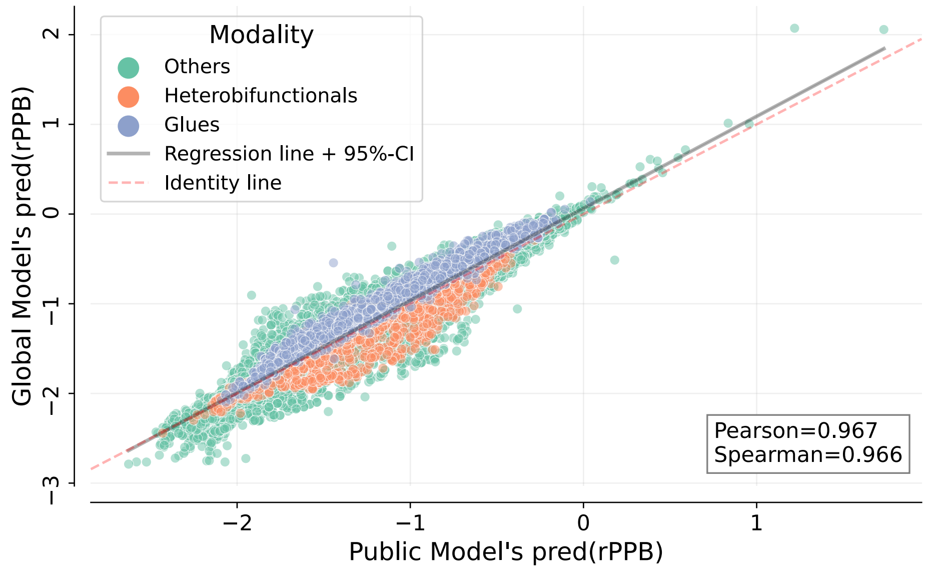

Modality
Others
Heterobifunctionals
Glues
Regression line + 95%-CI
Identity line
Pearson=0.967
Spearman=0.966
Global Model's pred(rPPB)
Public Model's pred(rPPB)

(I)

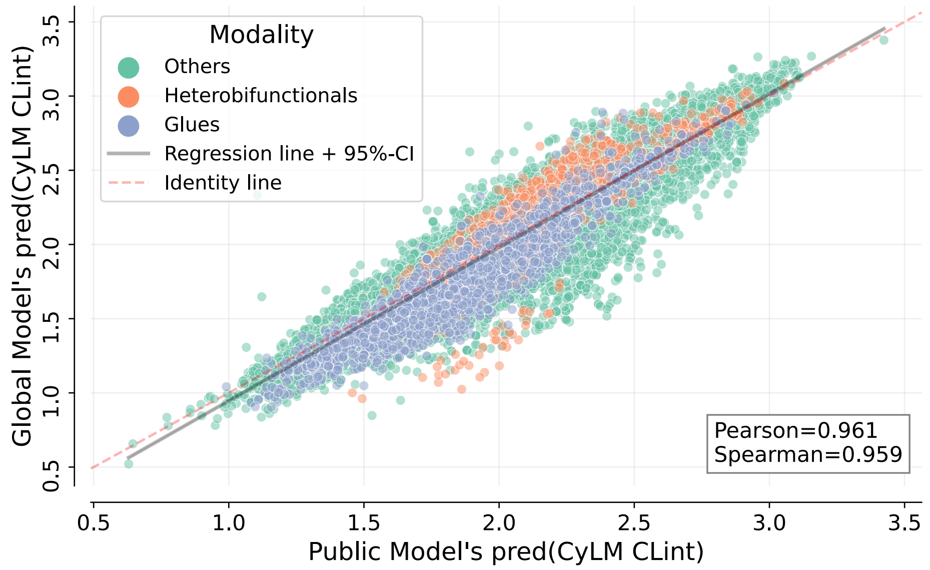

Modality
Others
Heterobifunctionals
Glues
Regression line + 95%-CI
Identity line
Pearson=0.961
Spearman=0.959
Global Model's pred(CyLM CLint)
Public Model's pred(CyLM CLint)

(J)

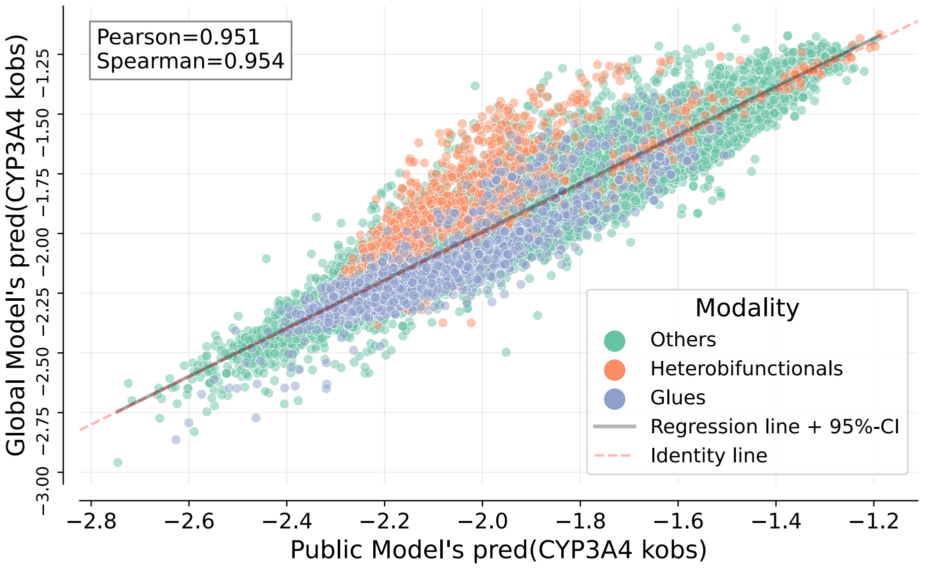
Pearson=0.951
Spearman=0.954
Modality
Others
Heterobifunctionals
Glues
Regression line + 95%-CI
Identity line
Global Model's pred(CYP3A4 kobs)
Public Model's pred(CYP3A4 kobs)
−3.00
−2.75
−2.50
−2.25
−2.00
−1.75
−1.50
−1.25
−2.8
−2.6
−2.4
−2.2
−2.0
−1.8
−1.6
−1.4
−1.2

(K)

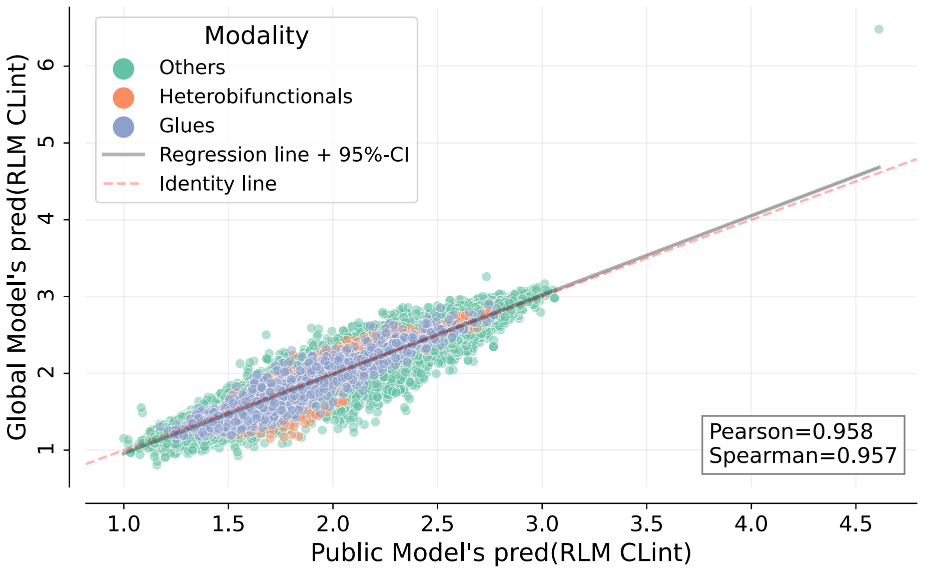
Modality
Others
Heterobifunctionals
Glues
Regression line + 95%-CI
Identity line
Pearson=0.958
Spearman=0.957
Global Model's pred(RLM CLint)
Public Model's pred(RLM CLint)
1
2
3
4
5
6
1.0
1.5
2.0
2.5
3.0
3.5
4.0
4.5

(L)

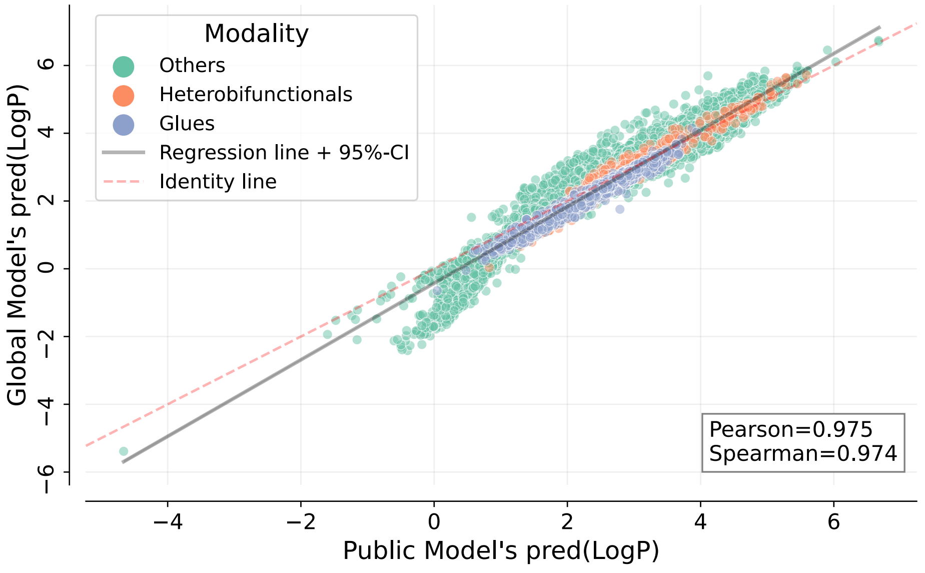
Modality
Others
Heterobifunctionals
Glues
Regression line + 95%-CI
Identity line
Pearson=0.975
Spearman=0.974
Global Model's pred(LogP)
Public Model's pred(LogP)
−6
−4
−2
0
2
4
6
−4
−2
0
2
4
6

(M)

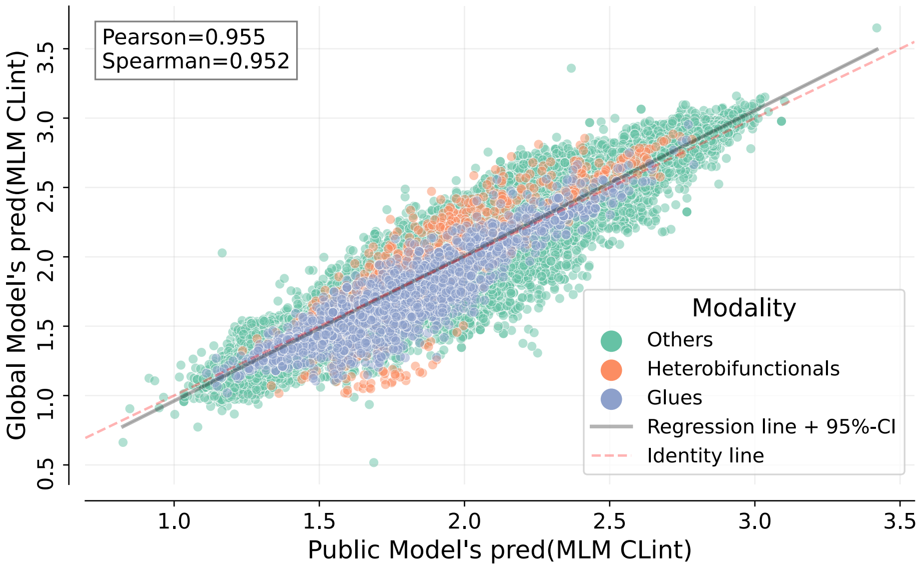

Pearson=0.955
Spearman=0.952
Modality
Others
Heterobifunctionals
Glues
Regression line + 95%-CI
Identity line
Global Model's pred(MLM CLint)
Public Model's pred(MLM CLint)

(N)

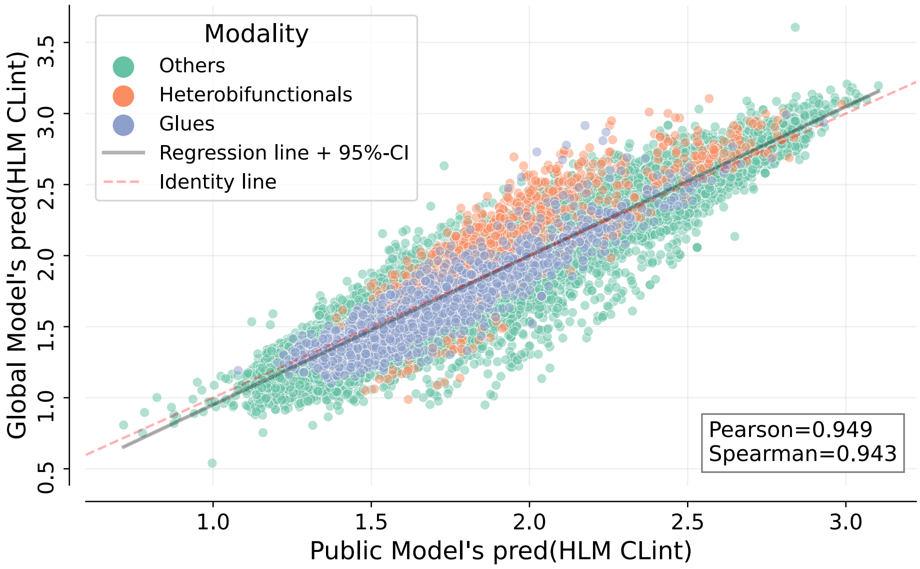

Modality
Others
Heterobifunctionals
Glues
Regression line + 95%-CI
Identity line
Pearson=0.949
Spearman=0.943
Global Model's pred(HLM CLint)
Public Model's pred(HLM CLint)

(O)

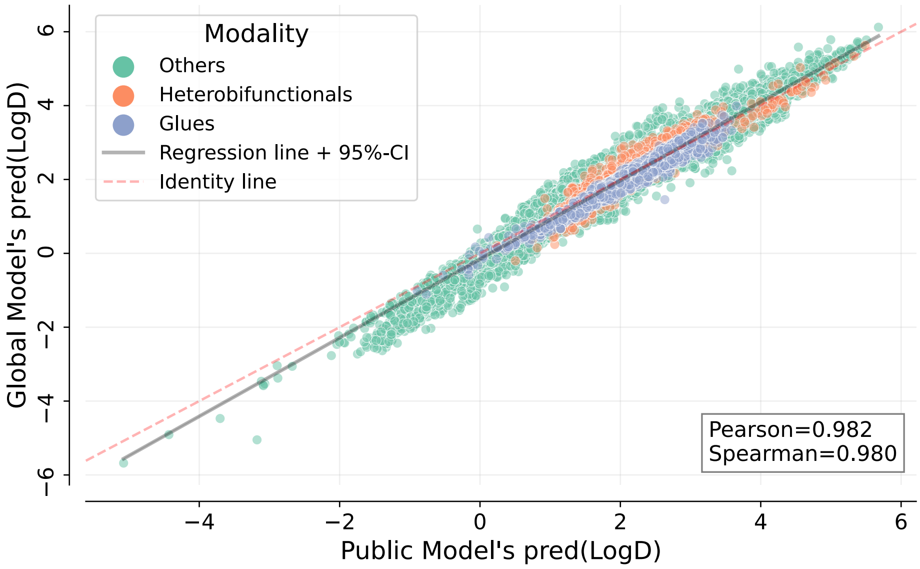

Modality
Others
Heterobifunctionals
Glues
Regression line + 95%-CI
Identity line
Pearson=0.982
Spearman=0.980
Global Model's pred(LogD)
Public Model's pred(LogD)

Figure S4. Original and surrogate models' predictions. Shown are the predictions from the multi-task graph neural network (MT-GNN) models derived with in-house data (original model) and surrogate data (public model). Shown are (A) CYP2C9 IC_{50} (pIC_{50}); (B) CYP2D6 IC_{50} (pIC_{50}); (C) cynoPPB ($LogF_u$); (D) CYP3A4 IC_{50} (pIC_{50}); (E) hPPB ($LogF_u$); (F) DLM CL_{int} ($LogCL_{int}$); (G) LE-MDCK v2 P_{app} ($LogP_{app}$); (H) rPPB ($LogF_u$); (I) CynoLM CL_{int} ($LogCL_{int}$); (J) CYP3A4 k_{obs} ($Logk_{obs}$); (K) RLM CL_{int} ($LogCL_{int}$); (L) LogP; (M) MLM CL_{int} ($LogCL_{int}$); (N) HLM CL_{int} ($LogCL_{int}$); (O) LogD. Assays are described in Table 1. Source data are provided as a Source Data file.

Table S1. Class distributions. Reported are the percentage of molecules categorized into each risk category for the training and test sets for the assays where classification performance is evaluated (LE-MDCK v2 P_{app}, RLM CL_{int}, HLM CL_{int}, CYP3A4 k_{obs}, and CYP3A4 IC_{50}). Assays are described in Table 1.

**Property (Short name)**	**Subset**	**Low risk**	**Medium Risk**	**High risk**
**LE-MDCK v2 P_{app}**	Training	17%	13%	70%
	Test	18%	14%	68%
**RLM CL_{int}**	Training	53%	22%	25%
	Test	60%	20%	20%
**HLM CL_{int}**	Training	67%	19%	14%
	Test	69%	17%	14%
**CYP3A4 k_{obs}**	Training	46%	29%	25%
	Test	45%	34%	21%
**CYP3A4 IC_{50}**	Training	63%	27%	10%
	Test	77%	18%	5%

Table S2. Models' architectures. Hyperparameters and architecture details that differ between the models are reported, including the number (#) of message passing steps, hidden units both in the message passing network (MPN) and feed-forward deep neural network (DNN), # hidden layers in DNN, dropout rate, # epochs, and ensemble size.

**Model**	**# Message passing steps**	**# Hidden layers in DNN**	**# Hidden units**	**Dropout**	**# Epochs**	**Ensemble size**
Permeability	3	2	300	0	30	5
Clearance	3	2	300	0	30	10
Binding/Lipophilicity	4	3	2100	0.3	20	10
CYP inhibition	6	3	400	0.1	30	10

Table S3. Global models' training set size. Reported are the total number of training set compounds for all the tasks included in the global models, as well as the number of glues and heterobifunctionals.

Property	Model	Glues	Heterobifunctionals	All
**LE-MDCK v1 P_{app}**	Permeability	1265	1274	47642
**LE-MDCK v2 P_{app}**	Permeability	1404	1608	20041
**PAMPA**	Permeability	53	170	138158
**Caco-2**	Permeability	3	0	31254
**MDCK-MDR1**	Permeability	113	200	10375
**RLM CL_{int}**	Clearance	2269	2326	185809
**HLM CL_{int}**	Clearance	2127	2206	123072
**MLM CL_{int}**	Clearance	1968	2108	93684
**DLM CL_{int}**	Clearance	201	108	9927
**CynLM CL_{int}**	Clearance	638	110	8326
**MinipigLM CL_{int}**	Clearance	0	3	686
**rPPB**	Binding/ Lipophilicity	74	232	11074
**hPPB**	Binding/ Lipophilicity	90	252	8365
**mPPB**	Binding/ Lipophilicity	81	290	9850
**dPPB**	Binding/ Lipophilicity	15	77	2192
**cynoPPB**	Binding/ Lipophilicity	47	63	753
**HSA**	Binding/ Lipophilicity	268	556	17028
**$f_{u,mic}$**	Binding/ Lipophilicity	0	31	782
**$f_{u,brain}$**	Binding/ Lipophilicity	4	0	819
**LogP**	Binding/ Lipophilicity	975	1680	26628
**LogD**	Binding/ Lipophilicity	957	1690	26484
**CYP3A4 k_{obs}**	CYP inhibition	384	1050	29431
**CYP3A4 IC_{50}**	CYP inhibition	318	1397	62570
**CYP2C9 IC_{50}**	CYP inhibition	261	809	34238
**CYP2D6 IC_{50}**	CYP inhibition	259	803	34333

Table S4. Training, fine-tuning, and test set sizes. Reported are the number of compounds in the training, fine-tuning, and test sets for the heterobifunctional compounds and compounds from all modalities. Note that models were only tested for heterobifunctionals, and fine-tuning strategy 2 only utilized heterobifunctional targeted protein degraders' data.

Property	Training set		Fine-tuning 1		Fine-tuning 2	Test set
	Heterobifunctionals	All modalities	Heterobifunctionals	All modalities	Heterobifunctionals	Heterobifunctionals
LE-MDCK v2 P_{app}	2187	32155	625	13079	2233	283
RLM CL_{int}	2775	198364	475	13981	2801	145
HLM CL_{int}	2652	135540	470	13996	2676	147
CYP3A4 k_{obs}	1258	30884	330	3510	1380	72
CYP3A4 IC_{50}	1573	64627	251	2708	1648	55
