# Supplementary material for: Application of machine learning models for property prediction to targeted protein degraders
Source: Nat Commun. 2024 Jul 9;15:5764. doi: 10.1038/s41467-024-49979-3 (PMC11233499; doi:10.1038/s41467-024-49979-3)
Supplement: Supplementary file 3 — Description Additional Supplementary Files [file 41467_2024_49979_MOESM3_ESM.pdf]

Description of Additional Supplementary Files

File Name: Supplementary Data 1

Description: Surrogate data set with publicly available structures (including targeted protein degraders) and predicted properties. The dataset contains a total of 273,705 structures extracted from ChEMBL, ZINC and PROTACDB 2.0 and predictions for twenty-five properties from physicochemical and absorption, distribution, metabolism, and excretion (ADME) assays.

File Name: Supplementary Software 1

Description: Code to build the four multi-task graph neural network (MT-GNN) global models and obtain property predictions.
